# Evidence of validity of internal structure of the Functional Assessment of Chronic Illness Therapy-Spiritual Well-Being Scale (FACIT-Sp-12) in Brazilian adolescents with chronic health conditions

**DOI:** 10.3389/fpsyg.2022.991771

**Published:** 2022-09-26

**Authors:** Willyane de Andrade Alvarenga, Lucila Castanheira Nascimento, Flávio Rebustini, Claudia Benedita dos Santos, Holger Muehlan, Silke Schmidt, Monika Bullinger, Fernanda Mayrink Gonçalves Liberato, Margarida Vieira

**Affiliations:** ^1^Department of Maternal-Infant and Public Health Nursing, WHO Collaborating Centre for Nursing Research Development, University of São Paulo at Ribeirão Preto College of Nursing, Ribeirão Preto, Brazil; ^2^Centre for Interdisciplinary Research in Health, School of Nursing, Institute of Health Sciences, Universidade Católica Portuguesa, Porto, Portugal; ^3^Escola de Artes, Ciências e Humanidades, Universidade de São Paulo, São Paulo, Brazil; ^4^Department Health and Prevention, Institute of Psychology, University of Greifswald, Greifswald, Germany; ^5^Department of Medical Psychology, University Medical Center Hamburg-Eppendorf, Hamburg, Germany; ^6^Departamento de Educação Integrada à Saúde, Universidade Federal do Espírito Santo, Espírito Santo, Brazil

**Keywords:** adolescents, chronic disease, FACIT, spirituality, psychometric testing, validation, Brazil

## Abstract

This study explored the evidence of validity of internal structure of the 12-item Functional Assessment of Chronic Illness Therapy—Spiritual Wellbeing Scale (FACIT-Sp-12) in Brazilian adolescents with chronic health conditions. The study involved 301 Brazilian adolescents with cancer, type 1 diabetes mellitus, or cystic fibrosis. Exploratory Factor Analysis (EFA), Confirmatory Factor Analysis (CFA), and Item Response Theory (IRT) were used to test the internal structure. Reliability was determined with Cronbach’s Alpha and McDonald’s Omega. The EFA suggested a one-dimensional scale structure in contrast to the original 2-factor model or the 3-factor model which were not reproduced in the current CFA. All quality indicators for the EFA one-factor exceeded the required criteria (FDI = 0.97, EAP = 0.97, SR = 3.96 and EPTD = 0.96, latent GH = 0.90. and the observed GH = 0.85). The FACIT-Sp-12 for adolescents yielded strong evidence for a 1-factor model and with good reliability.

## Introduction

Spirituality has become an important issue in person-centered health care. An increasing number of studies have described its relationship with patient reported health outcomes, especially in the areas of oncology and mental health (Koenig, 2012; McLouth et al., 2021). Adolescents with life-threatening diseases or severe chronic conditions meet challenges in coping with their condition and spirituality may affect the psychological and behavioral adjustment (Damsma Bakker et al., 2018; Alvarenga et al., 2021). The role of spiritual wellbeing, however, is not well understood in young people, and the number of reliable and valid instruments to assess the construct in this age group is still limited (Cotton et al., 2010).

Spiritual wellbeing refers to a sense of meaning or purpose in life, inner peace, harmony, strength, and comfort from faith (Peterman et al., 2002). The 12-item Functional Assessment of Chronic Illness Therapy—Spiritual Wellbeing (FACIT-Sp-12) Scale (Peterman et al., 2002) is the most widely used scale to assess spiritual wellbeing in individuals with cancer and other chronic illnesses (Peterman et al., 2014). It is considered suitable for religious or non-religious people to assess the patient’s current spiritual state (Monod et al., 2011). FACIT-Sp-12 has also been used with adolescents, even though there is no evidence for its validity in this population (Cotton et al., 2009, 2012). Recently, the measure was adapted for Brazilian adolescents with chronic diseases (de Alvarenga et al., 2019). It was assumed that the cognitive and emotional development reached at this age enables respondents to experience and express spirituality (Cotton et al., 2010). However, the psychometric performance of this scale has been validated with Brazilian adults, it has not yet been evaluated with adolescents (Lucchetti et al., 2013).

The initial psychometric analysis of the FACIT-Sp-12 in adults with cancer and HIV supported two factors (meaning/peace, faith) (Peterman et al., 2002). However, recent studies with a sample of adults with cancer and other chronic illnesses have demonstrated a superiority in three-factor model (meaning, peace, faith) as compared to the two-factor model (Canada et al., 2008; Murphy et al., 2010; Peterman et al., 2014). Consensus regarding the factorial structure of the instrument has not yet been determined. No previous study has investigated the factorial validity of FACIT-Sp-12 in adolescents. Analysis of the FACIT-Sp-12 in the subgroup of Brazilian adolescents cast some doubt on the appropriateness of the two- or three-factor model, suggesting that additional studies with larger samples are required for the validation of the scale in this age group (de Alvarenga et al., 2019). Thus, the current study aimed to demonstrate evidence of the validity of internal structure FACIT-Sp-12 for Brazilian adolescents with chronic illness. Research questions were: (a) How well do the 2- and 3-factor models of the FACIT-Sp-12 fit the observed data? and (b) Does the FACIT-Sp-12 show sufficient reliability in Brazilian adolescents?

## Materials and methods

### Setting and sample

The total sample consisted of 301 adolescents from 19 different states in Brazil. Inclusion criteria were: (1) age from 12 to 17 years, (2) diagnosis of chronic illness (cancer, type 1 diabetes mellitus, or cystic fibrosis), (3) regular outpatient or inpatient at clinical follow-up, and (4) ability to read/write Portuguese. Adolescents who had been reported to have intellectual disabilities in medical records or by health professionals were excluded. Recruiting more than 20 participants per item or a sample size of 300 to evaluate the dimensionality of the scale using EFA was considered appropriate (Costello and Osborne, 2005; Hair et al., 2018).

Convenience sampling was used to recruit participants at five hospitals with pediatric clinics (oncology, endocrine, or genetic diseases clinic) in Brazil. These were the University Hospital of the Medical School of Ribeirão Preto, Cancer Hospital of Barretos, Uberlândia Clinics Hospital, Diabetes Center of the Federal University of São Paulo, and Cystic Fibrosis Center of the Nossa Senhora da Glória Children’s Hospital. The study protocol was approved by the Brazilian Ethics Board (approval number #2.000.918). Potential participants received information about the study, and written consent was obtained from the adolescents and their legal guardians prior to the start of the study.

### Functional assessment of chronic illness therapy—Spiritual wellbeing-12 spiritual wellbeing scale

Demographic and clinical information, collected from case registries and patient self-reports included age, gender, type of chronic disease, level of education, region of origin, and religion.

FACIT-Sp-12 (Version 4), originally developed for adults, was adapted for adolescents in the Portuguese language (de Alvarenga et al., 2019). As a result, changes in the wording of some items were required which were recommended by a committee of experts on the basis of prior cognitive interviews with adolescents. The scale consists of 12 items formatted in a five-point Likert scale ranging from 0 = not at all to 4 = very much, with the exception of two negatively stated items (4 and 8) coded in a reverse manner (Peterman et al., 2002). The responses to the self-reported items refer to a 7-day recall period. The scores are added to generate a total score ranging from 0 to 48 (Bredle et al., 2011). With the highest scores representing better spiritual wellbeing. FACIT-Sp-12 was included in this study with the consent of the original authors of the scale at FACIT.org.

Participants completed the study instruments under the guidance of the primary investigator and one trained research assistant (undergraduate nursing student or nurse). There was no missing data.

### Statistical analysis

The data were analyzed by exploratory factor analysis (EFA), confirmatory factor analysis (CFA), and Item Response Theory (IRT). The software SPSS 23, AMOS 23, and Factor 10.8 were used.

#### Exploratory factor analysis

Dimensionality testing was performed with the Robust Parallel Analysis (RPA) using the optimal implementation of Parallel Analysis (PA) with the minimum rank factor analysis and permutation with 500 random correlations. The robustness of the test was determined from the association of a bootstrap with a sample extrapolation to 5,000 cases. The estimation of the polychoric matrix was performed using the Bayes Modal Estimation (Choi et al., 2011). The factors were extracted using the Robust Unweighted Least Squares (RULS) technique, which reduces the matrix residues. Promax rotation was considered in case of identification of multiples factors, indicating multidimensionality. For each factor extracted, a set of one-dimensional evaluation indicators was adopted (Ferrando and Lorenzo-Seva, 2018), namely, Unidimensional Congruence > 0.95 (UNICO), Explained Common Variance > 0.80 (ECV) (Quinn, 2014), and Mean of Item Residual Absolute Loadings < 0.30 (MIREAL).

#### Item response theory

The Multidimensional Normal-Ogive Graded Response Model of Reckase’s parameterization technique (Reckase, 1985) was used to identify a multidimensional polytomous structure, since the models published in the literature for FACIT-Sp-12 were multidimensional. The item’s discrimination index, which measures the strength of the association between the item and the latent variable and is equivalent in meaning to factorial loads of the EFA was used to complement the EFA (Jordan and Spiess, 2019).

#### Factorial quality parameters

The explained variance of factors in the instrument should be around 60% (Hair et al., 2018). An initial factorial load of at least 0.30, better 0.50 is recommended in a sample of over 300 individuals (Hair et al., 2018); commonalities must have values above 0.40 (Costello and Osborne, 2005). Decisions about the retention or removal of an item from the model depends on the magnitude of the commonality, factor loadings, sample size, degree to which the item represents the factor (Gaskin and Happell, 2014) and the lack of cross-loading (Hair et al., 2018).

#### Adjustment indices in the confirmatory factor analysis

Models in the confirmatory analysis (Hair et al., 2018) took into account parameters that considered the number of participants, and requirements to employ adjustment parameters. The parameters adopted for the cutoff of goodness of fit were based on the study by Sivo et al. (2006). Minimum indexes for adequacy: NNFI (Non-Normed Fit Index ≥0.97); CFI (Comparative Fit Index ≥ 0.97); GFI (Goodness Fit Index ≥ 0.93); AGFI (Adjusted Goodness Fit Index ≥ 0.91); RMSEA (Root Mean Square Error of Approximation ≤ 0.07), and RMSR (Root Mean Square of Residuals ≤ 0.10).

#### Reliability

The Cronbach’s Alpha and McDonald’s Omega indicators were used to determine the scale reliability with an acceptance threshold of > 0.70 (Hair et al., 2018).

#### Replicability and quality of the factorial solution

The construct’s replicability was assessed by the Generalized G-H Index requiring an index greater than 0.80. For the quality of factor scores estimates, the factor determinacy index (FDI) was used to identify at adequacy with estimate values higher than 0.90, EAP marginal reliability (> 0.80), sensibility ratio (SR > 2), and expected percentage of true differences (EPTD > 90%) (Rodriguez et al., 2016). Complementary indexes were applied to exclude the likelihood of primary indexes (goodness-of-fit) to overestimate the factorial solution based on low-quality items in the CFA model (Ferrando and Lorenzo-Seva, 2018).

## Results

### Sample

A total of 301 adolescents, median age 15 years, with cancer (46.5%), type 1 diabetes mellitus (35.5%), and cystic fibrosis (17.9%) participated in this study (see Table 1 for sample characteristics). Among them 82.3% adhered to a religion, and 93.1% considered spirituality very or somewhat important.

**TABLE 1:** Characteristics of adolescents (*N* = 301).

Variable		M/Md/SD	*n*	%
Age (12–17 years)		14.5/15/3.3		
Time since diagnosis	<1		86	28.6
	1–9 years		157	49.2
	10–17 years		58	22.2
Gender	Male		156	51.8
	Female		145	48.2
Chronic disease	Cancer		140	46.5
	Type 1 diabetes mellitus		107	35.5
	Cystic fibrosis		54	17.9
Region of origin in Brazil	North		32	10.7
	Northeast		6	2.0
	Midwest		19	6.3
	Southeast		241	80.1
	South		3	1.0
Level of education	≤ Middle school		169	56.2
	High school		132	43.8
Denomination	Catholic		136	45.2
	Protestants		94	31.2
	Spiritist		12	4.0
	Other religion		6	1.9
	Not religious but spiritual (believes in something)		46	15.3
	Neither religious nor spiritual (believes in nothing)		2	0.7
	Atheist		5	1.7
Importance of spirituality	Important		179	59.5
	Somewhat important		101	33.6
	Not very important		14	4.7
	Not at all important		7	2.3

### Factorial structure

The original 2-factor model (Peterman et al., 2002) was established as an initial model for extraction. The result of the dimensionality testing, based on eigenvalue values greater than one, identified two factors with an explained variance of 60%. However, the RPA, a more accurate method, indicated the existence of only 1-factor with an explained variance of 59.1%. The result of the parallel analysis with only 1-factor is confirmed by the values of UNICO = 0.938, nearly reaching the one-dimensional limit of 0.95. The results of ECV = 0.811 and MIREAL = 0.274 reaffirmed that the items of the instrument are best represented in a one-dimensional model. To cross check this finding, we performed an additional analysis of dimensionality using the Mininum Average Partial (MAP) method (Velicer, 1976). The average partial correlation of MAP result = 0.048 (*p* < 0.001) also strongly supported the one-factor solution. In studies in which there is a divergence between the number of factors found, the use of more than one dimensionality technique is recommended (Bandalos, 2018).

In compliance with the recommendation to examine the one dimensional vs. the multidimensional models of the FACIT-Sp-12, we chose to analyze the factorial loads, commonality, and item discrimination in the three models (see Table 2): 1-factor model, the original 2-factor model (Peterman et al., 2002), and the recently published 3-factor model (Canada et al., 2008; Murphy et al., 2010; Peterman et al., 2014).

**TABLE 2:** Factor loading, communalities and item discrimination of one-, two-, and three-factor models of the FACIT-Sp-12.

Original FACIT-Sp-12 items (*FACIT-Sp-12 for Brazilian adolescents*)	1-factor model			2-factor model				3-factor model				
	λ factor 1	h^2^	“a”	λ factor 1	λ factor 2	h^2^	MDISC	λ factor 1	λ factor 2	λ factor 3	h^2^	MDISC
01–I feel peaceful *(Sinto-me em paz)*	0.69	0.48	0.96	0.76		0.56	1.15	0.67			0.55	1.02
02—I have a reason for living *(Tenho uma razão para viver)*	0.75	0.56	1.13	0.68		0.58	1.07	0.59			0.57	0.95
03—My life has been productive *(A minha vida tem sido proveitosa)*	0.60	0.36	0.75	0.56		0.38	0.72	0.45			0.37	0.61
**04—I have trouble feeling peace of mind** ***(Tenho dificuldade em sentir paz interior)***	0.48	0.23	0.55	0.37		0.23	0.46				0.25	0.41
05—I feel a sense of purpose in my life *(Sinto que a minha vida tem um objetivo)*	0.70	0.48	0.98	0.53		0.47	0.80	0.38			0.47	0.67
06—I am able to reach down deep into myself for comfort *(Sou capaz de encontrar conforto dentro de mim mesmo/a)*	0.65	0.42	0.86	0.76		0.52	1.10	0.92			0.47	1.55
07—I feel a sense of harmony within myself *(Sinto-me em harmonia comigo mesmo/a)*	0.72	0.52	1.03	0.88		0.68	1.57	0.83			0.63	1.49
**08—My life lacks meaning and purpose** ***(Faltam sentido e objetivo em minha vida)***	0.65	0.43	0.86	0.63		0.45	0.86			1.06	0.68	†
09—I find comfort in my faith or spiritual beliefs *(Encontro conforto na minha fé ou crenças espirituais)*	0.64	0.41	0.83		0.93	0.79	2.04		0.95		1.00	2.12
10—I find strength in my faith or spiritual beliefs (*Encontro força na minha fé ou crenças espirituais)*	0.69	0.48	0.97		0.99	0.92	3.59		1.01		0.92	3.72
11—My illness has strengthened my faith or spiritual beliefs *(A minha doença tem fortalecido a minha fé ou crenças espirituais)*	0.63	0.40	0.82		0.61	0.50	0.88		0.61		0.50	0.90
12—I know that whatever happens with my illness, things will be okay *(Independentemente do que acontecer com a minha doença, eu sei que as coisas ficarão bem)*	0.61	0.37	0.77	0.33	0.36	0.37	0.62	0.35	0.37		0.38	0.66

Bold—items with reversed scoring; λ—loading factor; h^2^—communalities; “a”;—item discrimination; MDISC, multidimensional discrimination index; ^†^violation of the discrimination.

In the 1-factor model, the factor loadings were between 0.48 and 0.75, with the only one below 0.60 being item 4. Factor loads above 0.50 indicate excellent configuration (Hair et al., 2018). Commonalities ranged between 0.23 and 0.56. However, items 4 (0.23), item 3 (0.36), and item 12 (0.37) were below the recommendation (> 0.40) (Hair et al., 2018). The item discrimination was between 0.55 and 1.13, and again only item 4 (0.55) was below the acceptability threshold of 0.65 (Baker, 2001). For the one-dimensional model, IRT conformity was tested with the Graded Response Model, which also indicated the possibility of removing item 4 from the model. Therefore, the model was tested without item 4, yet removal of this item did improve the model so that it was retained.,

In the 2-factor model, where the first dimension is composed of items 1–8, and the second dimension of items 9–12, the factor loadings were between 0.33 and 0.88, with two items (4 and 12) ranging below the critical values of 0.50. Another problem appeared in the two-dimensional model, with item 12 cross-loadings with both dimensions, thus violating the principle that each item should significantly load on only one dimension/factor (Hair et al., 2018). Item 12 presented factorial loads below the quality criterion, namely 0.33 in dimension 1 and 0.36 in dimension 2. Since he scores ranged from 0.23 to 0.92 in the commonalities. In the IRT analysis using the Reckase technique, items 4 and 12 were below the limit. Despite of their poor performance items 3, 4, and 12 were retained in this model at this stage.

In the 3-factor model, the dimension/factor 3 is composed of only 1 item (8), and the factor load is greater than 1, which presents a Heywood case (Costello and Osborne, 2005). The same occurred with Item 10 of dimension 2 Item 12 continued to show double saturation, and item 4 had no significant factor load an any of the dimensions. In addition, 5 of the 12 items did not reach the minimum for item discrimination in the 3-factor model.

**FIGURE 1:**
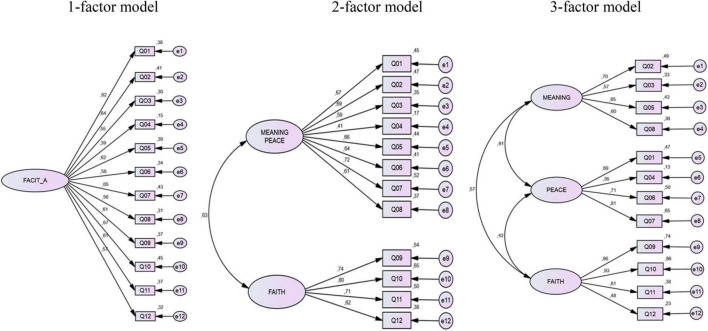
Pathway—measurement model for the one-, two- and three-factor structures of the FACIT-Sp-12.

As the 2-factor and 3-factor model did not fit in the EFA, CFA was performed on all three models in the next step. For CFA the 2 and 3 factors were used according to the previous studies (Peterman et al., 2002, 2014; Canada et al., 2008; Murphy et al., 2010). As in Figure 1 shows, the factorial loads of the one-dimensional model ranged from 0.39 to 0.67, and the *R*^2^ from 0.15 to 0.45. For both indicators, the lowest values were found for item 4. In the two-dimensional model, the loads ranged from 0.41 to 0.74, and *R*^2^ from 0.17 to 0.65, with item 4 demonstrating the lowest factor load and item 3 the lowest R^2^. The correlation between factors in the two-dimensional model was 0.63. In the three-dimensional model, the loads ranges between 0.36 and 0.93, and the *R*^2^ from 0.13 to 0.86, again with the floor values for item 4. In the three-dimensional model, factor loads for items 9 and 10 were high, namely 0.86 and 0.93. The correlations between factors were high for Meaning with -Peace (*r* = 0.91), Peace-Faith (*r* = 0.42), and Meaning-Faith (*r* = 0.57). The extremely high correlation coefficients between Meaning-Peace indicate that the dimensions measuring the same concept.

Synthesis of models is provided in Table 3. The goodness-of-fit values increase also with the number of dimensions. However, this does not indicate an overall superiority of two-dimensional and three-dimensional models as compared to the one-dimensional model.

**TABLE 3:** Comparison of models of the FACIT-Sp-12 for one-, two-, and three-factor structures.

	Index	Technique	1-factor	2-factor	3-factor
Exploratory factor analysis	Adequacy of the correlation matrix^†^	Determinant of the matrix	0.0006		
		Bartlett’s test of sphericity	1465.5 (df = 66)*		
		KMO (Kaiser-Meyer-Olkin)	0.86 (95% CI 0.848–0.901)		
	Dimensions (parallel analysis—PA)		1		
	Variance explained by eigenvalues		47.80%	60.00%	67.30%
	Variance explained (PA)^†^		59.10%		
	Polychoric correlations (rp =)^†^		0.25–0.87		
Dimensionality	One-dimensional congruence (UNICO)		0.96		
	Explained common variance (ECV)		0.81		
	Mean of item residual absolute loading (MIREAL)		0.27		
Confirmatory factor analysis	Robust mean and variance-adjusted chi square (df = 54)		260.33*	76.42	51.40
	Non-normed fit index (NNFI)		0.93	0.98	0.99
	Comparative fit index (CFI)		0.95	0.99	0.99
	Goodness-of-fit index (GFI)		0.97	0.99	0.99
	Adjusted goodness of fit index (AGFI)		0.96	0.98	0.99
	Root mean square error of approximation (RMSEA)		0.11	0.05	0.04
	Root mean square of residuals (RMSR)		0.10	0.04	0.03
Reliability	Standardized Cronbach’s Alpha^†^		0.89		
	McDonald’s Omega^†^		0.90		
	Construct reliability—GH latent index (>0.80)		0.90	0.89–0.90	0.91–0.95–0.99
Quality of the factorial solution^‡^	Factor determinacy index (FDI > 0.90)		0.97	0.96–0.92	0.95–0.93–0.81
	EAP marginal reliability (>0.80)		0.94	0.92–0.86	0.91–0.86–0.65
	Sensibility ratio (SR>2)		3.96	3.52–2.49	3.28–2.54–1.38
	Expected percentage of true differences (EPTD > 90%)		96%	95.5–96.3%	95.3–96.6–89.3%

^†^The values are the same for all three models, as the technique indicates one-dimension as significant; *p < 0.001; ^‡^value for each dimension.

### Reliability, quality of the factorial solution, and replicability of the 1-factor model

Using quality threshold criteria for the Cronbach’s alpha (0.89) and the McDonald’s Omega (0.90), reliability reached adequate levels for the three models. All quality indicators of the factorial solution have important levels and higher than the minimum limit required for the quality of the solution: FDI = 0.97, EAP = 0.97, SR = 3.96, and EPTD = 0.96. The GH index with its threshold of = 0.80) measures the model’s ability to maintain its stability in other contexts and samples. The latent G-H Index was 0.90, and the observed G-H Index was 0.85 and therefore close to each other in the 1- factor model suggesting stability (Ferrando and Lorenzo-Seva, 2018) of the 1-factor model.

## Discussion

We evaluated the psychometric properties of the FACIT-Sp-12 in a sample of 301 Brazilian adolescents with chronic health conditions. The FACIT-Sp-12 in this sample showed evidence of validity. The factorial structure was acceptable, and the EFA suggested one-dimensionality with quality indicators superior to the alternative 2 and 3 dimensional models. Several complementary indexes were used to assess the quality of the factorial solution because the use of only one index does not guarantee that the factorial solution is adequate (Ferrando and Lorenzo-Seva, 2018).

The EFA demonstrated only one factor through parallel analysis, although the eigenvalues for EFA had suggested two factors. This is a classic problem in psychometrics when using eigenvalues which have been shown to be fragile by overestimating the number of dimensions (Horn, 1965) and by failing to adjust the model due to the effect of sample error as performed in parallel analysis (Hayton et al., 2004). The adoption of an “eigenvalue greater than one criterion” to determine the number of factors only contributes to the development of inaccurate measures and inefficient concepts (Patil et al., 2008). None of the studies with FACIT-Sp-12 used parallel analysis, which has been considered one of the most robust techniques for testing dimensionality (Lim and Jahng, 2019).

In the one-dimensional model, the explained variance and factorial loads were considered good as opposed to the two-dimensional and three-dimensional model. The three-dimensional model appeared to be the most problematic of all. The dimension/factor 3 is composed of only 1 item (item 8), a which presented a Heywood case. Several problems detected in the current study in relation to the 3-factor model have also been found in other studies (Canada et al., 2008; Murphy et al., 2010). In one study Item 8 presented double saturation between Meaning and Peace, item 4 and item 10—Heywood case- presented a factor load of 1.00 and 1.01, respectively (Canada et al., 2008). In another study (Murphy et al., 2010), item 10 had a factorial load equal to 1; there were covariance error controls between items 4 and 8 and crossing of item 12 for Meaning and Peace with a factorial load below 0.50.

Other problems appeared in the CFA path diagram because covariance error controls were performed on the model between item 4 for items 1 and 8 (Canada et al., 2008). This type of control is not a serious violation, as it is limited to up to 2 covariance errors for every 15 items (Hair et al., 2018). However, these controls demonstrate that the instabilities associated with these items were controlled. Notable is that the original version of the instrument did not reach the necessary quality indicators without these controls. The pathways diagram also indicated a cross-loading saturation of item 12 on the Peace and Faith dimension, indicating that item 12 may be associated with both Faith and Peace (Canada et al., 2008; Murphy et al., 2010). The original study did not explain the reason for this crossover, with item 12 being the only one to have a load below 0.50 in CFA with 3 factors (Canada et al., 2008).

Items 4, 8, and 12 are the basis of the instabilities found in the 2-factor model and the 3-factor model in the study with adolescents. It should be noted that items 4 and 8 underwent score reversal and not coincidentally are the ones that presented numerous problems. In the Spanish version, these items again had the lowest factor loads (Galiana et al., 2016). In the Norwegian version, the initial extraction presented 4 dimensions, however, with many cross-loading (Haugan, 2015). Hence, the author decided to test the version with 2 and 3 dimensions. In the two-dimensional version, item 3 had no significant factor load, and items 9–12 revealed cross-loadings for dimensions 2 and 3. In CFA, items 3, 4, and 12 have loads below 0.50, only 0.22 for item 12. In the French version (Agli et al., 2017), in addition to the removal of item 3 that was not part of the model presented, the items are in a different position from the proposal with 3 dimensions (Canada et al., 2008). In the Greek version, with 3 dimensions, there was double saturation in items 2, 5, and 12 (Fradelos et al., 2016). Another study presented another configuration with item 3 demonstrating double saturation, while item 12 became part of the Peace dimension instead of Faith (Bai and Dixon, 2014).

Prior validity studies of FACIT-Sp-12 used Principal Components Analysis (PCA) as an extraction technique for the EFA, even though there is extensive literature indicating that PCA is not an EFA (Gorsuch, 1990; Widaman, 1993). The Common Factor Analysis (CFA—used in our study) is superior and more accurate than PCA (Fabrigar and Wegener, 2012). Several issues are to be considered in the differentiation between the techniques (Fabrigar and Wegener, 2012): (a) researchers usually assume that PCA is similar to EFA, which is not correct; (b) PCA models do not distinguish between common and unique variances; (c) CFA analysis assumes that the latent variable is the cause of the measured variables (items) which differs from the PCA; (d) PCA does not produce results similar to the CFA; and (e) in general CFA is preferable if the objective of the study is to identify a latent variable for the construction of a theory or to create an instrument.

In addition to the problems of techniques substantiation, the application of PCA caused an overestimation of the variance explained with 16.4% between the models and produced higher levels of factor loads and commonality, precisely because it partitions the single variance of the common variance (Costello and Osborne, 2005). de Winter and Dodou (2016) compared PCA with CFA, and their results show that PCA loadings correlated weakly with the true factor loadings for under-extraction, over-extraction, and heterogeneous loadings within factors. The pattern of differences between CFA and PCA was consistent across sample sizes, levels of loadings, main axis factoring vs. maximum likelihood factor analysis, and blind vs. target rotation.

Thus, the validity studies of FACIT-Sp-12 that adopted the eigenvalue greater than one and PCA criteria imply that the dimensions, explained variance, factorial loads, and commonality are overestimated. The adoption of PCA in studies that seek to demonstrate the validity of instruments through EFA was criticized by several authors who found that more than 50% of the studies still applied PCA despite the limitations of this technique for this purpose (Gaskin and Happell, 2014; Izquierdo et al., 2014).

Another aspect that may have affected the factorial analysis but was so far not considered in the published literature is item reversal. Guidelines for item writing have pointed out that negatively framed items, reverse wording, and inversion of scores, in the construction of scales results in systematic problems for statistical analysis (Ferrando and Lorenzo-Seva, 2018). It is essential that the construction of the scale levels occurs in increasing order in order to provide stable scores (Hamby and Levine, 2016).

This study has limitations related to sample composition and selectivity. The results of this study may differ in adolescents who are not chronically ill, in participants from other countries, or persons recruited from non-hospital settings, or adults, because the factorial structure of FACIT-Sp-12 with these samples were not examined (Lucchetti et al., 2013). The participating Brazilian adolescents were predominantly Christian, but if analyzes were conducted with adolescents who did not identify themselves as religious, the invariance of this scale could be assured. In addition, analyses of invariance of the scale’s factorial structure need to be verified between age groups and chronic disease types (cancer, diabetes mellitus, and cystic fibrosis).

The results of this study provide important new insights into the psychometric properties of FACIT-Sp-12 in a Brazilian adolescent population. This is the first comparative psychometric evaluation of FACIT-Sp-12 using three statistical approaches (EFA, CFA, and IRT) and additional multiple dimensionality testing techniques, such as the adoption of RPA and MAP based on the extraction model of common factors. The scale showed strong evidence of construct validity according to the extensive testing carried out in our study which applies various techniques and examined different indexes. This is also the first study suggesting a one-dimensional structure for the FACIT-Sp-12 and the use of a single summary score. This score can be easily calculated and can allow healthcare providers to assess spiritual wellbeing in Brazilian adolescents with chronic illnesses as well as to examine the effectiveness of interventions oriented toward spiritual wellbeing. The results of this research may enrich our understanding of the dimensions of spiritual wellbeing which maybe more strongly intertwined and psychometrically inseparable than originally assumed.

## Data availability statement

The original contributions presented in this study are included in the article/supplementary material, further inquiries can be directed to the corresponding author/s.

## Ethics statement

The studies involving human participants were reviewed and approved by the Ethics Board from Ribeirão Preto College of Nursing of University of São Paulo (approval number # 2.000.918). Written informed consent to participate in this study was provided by the participants’ legal guardian/next of kin.

## Author contributions

WA, LN, FR, CS, HM, and SS contributed to study conception and design, data acquisition, data analysis, and interpretation. MB, FL, and MV contributed to article writing and critical review of relevant intellectual content. LN and MV supervised the project. All authors contributed to the article and approved the submitted version.

## References

[B1] Agli O. Bailly N. Ferrand C. (2017). ‘Validation of the functional assessment of chronic illness therapy—spiritual well-being (FACIT-Sp12) on french old people’. *J. Relig. Health* 56 464–476. 10.1007/s10943-016-0220-0 26976133

[B2] Alvarenga W. A. Machado J. R. Leite A. C. A. B. Caldeira S. Vieira M. da Rocha S. S. (2021). ‘Spiritual needs of Brazilian children and adolescents with chronic illnesses: A thematic analysis’. *J. Pediatr. Nurs*. 60:e39–e45. 10.1016/j.pedn.2021.02.020 33648836

[B3] Bai M. Dixon J. K. (2014). ‘Exploratory factor analysis of the 12-item functional assessment of chronic illness therapy–spiritual well-being scale in people newly diagnosed with advanced cancer’. *J. Nurs. Meas.* 22 404–420. 10.1891/1061-3749.22.3.404 25608428

[B4] Baker F. B. (2001). *The Basics of Item Response Theory*, 2nd Edn. Washington, DC: ERIC Publications.

[B5] Bandalos D. L. (2018). *Measurement Theory and Applications for the Social Sciences*, 1st Edn. New York, NY: The Guilford Press.

[B6] Bredle J. M. Salsman J. M. Debb S. M. Arnold B. J. Cella D. (2011). ‘Spiritual well-being as a component of health-related quality of life: the functional assessment of chronic illness therapy—spiritual well-being scale (FACIT-Sp)’. *Religions* 2 77–94. 10.3390/rel2010077

[B7] Canada A. L. Canada A. L. Murphy P. E. Fitchett G. Peterman A. H. Schover L. R. (2008). ‘A 3-factor model for the FACIT-Sp’. *Psychooncology* 17 908–916. 10.1002/pon.1307 18095260

[B8] Choi J. Kim S. Chen J. Dannels S. (2011). ‘A comparison of maximum likelihood and bayesian estimation for polychoric correlation using monte carlo simulation’. *J. Educ. Behav. Stat.* 36 523–549. 10.3102/1076998610381398

[B9] Costello A. B. Osborne J. W. (2005). ‘Best practices in exploratory factor analysis: Four recommendations for getting the most from your analysis’. *Pract. Assess. Res. Eval.* 10:7.

[B10] Cotton S. Grossoehme D. Rosenthal S. L. McGrady M. E. Roberts Y. H. Hines J. (2009). ‘Religious/Spiritual coping in adolescents with sickle cell disease: A pilot study’. *J. Pediatr. Hematol. Oncol.* 31 313–318. 10.1097/MPH.0b013e31819e40e3 19415008 PMC2749498

[B11] Cotton S. McGrady M. E. Rosenthal S. L. (2010). ‘Measurement of religiosity/spirituality in adolescent health outcomes research: trends and recommendations’. *J. Relig. Health* 49 414–444. 10.1007/s10943-010-9324-0 20127172 PMC2917535

[B12] Cotton S. Weekes J. C. McGrady M. E. Rosenthal S. L. Yi M. S. Pargament K. (2012). ‘Spirituality and religiosity in urban adolescents with asthma’. *J. Relig. Health* 51 118–131. 10.1007/s10943-010-9408-x 20924680 PMC3090716

[B13] Damsma Bakker A. van Leeuwen R. R. Roodbol P. F. (2018). ‘The spirituality of children with chronic conditions: A qualitative meta-synthesis’. *J. Pediatr. Nurs.* 43:e106–e113. 10.1016/j.pedn.2018.08.003 30122453

[B14] de Alvarenga W. A. Nascimento L. C. Dos Santos C. B. Leite A. C. A. B. Mühlan H. Schmidt S. (2019). ‘Measuring spiritual well-being in brazilian adolescents with chronic illness using the FACIT-Sp-12: Age adaptation of the self-report version, development of the parental-report version, and validation’. *J. Relig. Health* 58 2219–2240. 10.1007/s10943-019-00901-y 31446605

[B15] de Winter J. C. F. Dodou D. (2016). ‘Common Factor Analysis versus Principal Component Analysis: A Comparison of Loadings by Means of Simulations’. *Commun. Stat. Simul. Comput.* 45 299–321. 10.1080/03610918.2013.862274

[B16] Fabrigar L. R. Wegener D. T. (2012). *Exploratory Factor Analysis.* New York, NY: Oxford University Press.

[B17] Ferrando P. J. Lorenzo-Seva U. (2018). ‘Assessing the quality and appropriateness of factor solutions and factor score estimates in exploratory item factor analysis’. *Educ. Psychol. Meas.* 78 762–780. 10.1177/0013164417719308 32655169 PMC7328234

[B18] Fradelos E. Tzavella F. Koukia E. Tsaras K. Papathanasiou I. V. Aroni A. (2016). ‘The translation, validation and cultural adaptation of functional assessment of chronic illness therapy - spiritual well-being 12 (facit-sp12) scale in greek language’. *Mater. Socio Med.* 28 229–234. 10.5455/msm.2016.28.229-234 27482168 PMC4949019

[B19] Galiana L. Sancho P. Oliver A. Tomás J. M. Calatayud P. (2016). ‘Envejecimiento y espiritualidad: Estructura factorial y fiabilidad de dos escalas’. *Rev. Esp. Geriatr. Gerontol.* 51 265–269. 10.1016/j.regg.2015.12.006 27068238

[B20] Gaskin C. J. Happell B. (2014). ‘On exploratory factor analysis: A review of recent evidence, an assessment of current practice, and recommendations for future use’. *Int. J. Nurs. Stud.* 51 511–521. 10.1016/j.ijnurstu.2013.10.005 24183474

[B21] Gorsuch R. L. (1990). ‘Common factor analysis versus component analysis: Some well and little known facts’. *Multivar. Behav. Res.* 25 33–39. 10.1207/s15327906mbr2501_3 26741966

[B22] Hair J. F. Babin B. J. Anderson R. E. William C. B. (2018). *Multivariate Data Analysis*, 8th Edn. Faridabad: International Thomson Business Press.

[B23] Hamby T. Levine D. S. (2016). ‘Response-scale formats and psychological distances between categories’. *Appl. Psychol. Meas.* 40 73–75. 10.1177/0146621615597961 29881037 PMC5978528

[B24] Haugan G. (2015). ‘The FACIT-Sp spiritual well-being scale: An investigation of the dimensionality, reliability and construct validity in a cognitively intact nursing home population’. *Scand. J. Caring Sci.* 29 152–164. 10.1111/scs.12123 24660831

[B25] Hayton J. C. Allen D. G. Scarpello V. (2004). ‘Factor retention decisions in exploratory factor analysis: A tutorial on parallel analysis’. *Organ. Res. Methods* 7 191–205. 10.1177/1094428104263675

[B26] Horn J. L. (1965). ‘A rationale and test for the number of factors in factor analysis’. *Psychometrika* 30 179–185. 10.1007/BF02289447 14306381

[B27] Izquierdo I. Olea J. Abad F. J. (2014). ‘Exploratory factor analysis in validation studies: Uses and recommendations.’. *Psicothema* 26 395–400. 10.7334/psicothema2013.349 25069561

[B28] Jordan P. Spiess M. (2019). ‘Rethinking the Interpretation of item discrimination and factor loadings’. *Educ. Psychol. Meas.* 79 1103–1132. 10.1177/0013164419843164 31619841 PMC6777065

[B29] Koenig H. G. (2012). ‘Religion, Spirituality, and Health: The Research and Clinical Implications’. *ISRN Psychiatry* 2012:278730. 10.5402/2012/278730 23762764 PMC3671693

[B30] Lim S. Jahng S. (2019). ‘Determining the number of factors using parallel analysis and its recent variants.’. *Psychol. Methods* 24 452–467. 10.1037/met0000230 31180694

[B31] Lucchetti G. Lucchetti A. L. G. de Gonçalves J. P. B. Vallada H. (2013). ‘Validation of the portuguese version of the functional assessment of chronic illness therapy–spiritual well-being scale (FACIT-Sp 12) among brazilian psychiatric inpatients’. *J. Relig. Health* 54 112–121. 10.1007/s10943-013-9785-z 24154632

[B32] McLouth L. E. Ford C. G. Pustejovsky J. E. Park C. L. Sherman A. C. Trevino K. (2021). ‘A systematic review and meta-analysis of effects of psychosocial interventions on spiritual well-being in adults with cancer’. *Psychooncology* 30 147–158. 10.1002/pon.5562 34602807 PMC8485897

[B33] Monod S. Brennan M. Rochat E. Martin E. Rochat S. Büla C. J. (2011). ‘Instruments measuring spirituality in clinical research: A systematic review’. *J. Gen. Intern. Med.* 26 1345–1357. 10.1007/s11606-011-1769-7 21725695 PMC3208480

[B34] Murphy P. E. Canada A. L. Fitchett G. Stein K. Portier K. Crammer C. (2010). ‘An examination of the 3-factor model and structural invariance across racial/ethnic groups for the FACIT-Sp: A report from the American Cancer Society’s Study of Cancer Survivors-II (SCS-II)’. *Psychooncology* 19 264–272. 10.1002/pon.1559 19367561

[B35] Patil V. H. Singh S. N. Mishra S. Donavan D. T. (2008). ‘Efficient theory development and factor retention criteria: Abandon the “eigenvalue greater than one” criterion’. *J. Bus. Res.* 61 162–170. 10.1016/j.jbusres.2007.05.008

[B36] Peterman A. H. Fitchett G. Brady M. J. Hernandez L. Cella D. (2002). ‘Measuring spiritual well-being in people with cancer: The functional assessment of chronic illness therapy?spiritual well-being scale (FACIT-Sp)’. *Ann. Behav. Med.* 24 49–58. 10.1207/S15324796ABM2401_06 12008794

[B37] Peterman A. H. Reeve C. L. Winford E. C. Cotton S. Salsman J. M. McQuellon R. (2014). ‘Measuring meaning and peace with the FACIT–Spiritual Well-Being Scale: Distinction without a difference?’. *Psychol. Assess.* 26 127–137. 10.1037/a0034805 24188147 PMC4081471

[B38] Quinn H. O. (2014). *Bifactor Models, Explained Common Variance (ECV), and the Usefulness of Scores from Unidimensional Item Response Theory Analyses.* Chapel Hill: University of North Carolina at Chapel Hill, 10.17615/t6ff-a088

[B39] Reckase M. D. (1985). ‘The Difficulty of Test Items That Measure More Than One Ability’. *Appl. Psychol. Meas.* 9 401–412. 10.1177/014662168500900409

[B40] Rodriguez A. Reise S. P. Haviland M. G. (2016). ‘Applying Bifactor Statistical Indices in the Evaluation of Psychological Measures’. *J. Pers. Assess.* 98 223–237. 10.1080/00223891.2015.1089249 26514921

[B41] Sivo S. A. Witta E. L. Willse J. T. (2006). ‘The Search for “Optimal” Cutoff Properties: Fit Index Criteria in Structural Equation Modeling’. *J. Exp. Educ.* 74 267–288. 10.3200/JEXE.74.3.267-288

[B42] Velicer W. F. (1976). ‘Determining the number of components from the matrix of partial correlations’. *Psychometrika* 41 321–327. 10.1007/BF02293557

[B43] Widaman K. F. (1993). ‘Common Factor Analysis Versus Principal Component Analysis: Differential Bias in Representing Model Parameters?’. *Multivar. Behav. Res.* 28 263–311. 10.1207/s15327906mbr2803_1 26776890

